# Branch site recognition by the spliceosome

**DOI:** 10.1261/rna.080198.124

**Published:** 2024-11

**Authors:** Jonas Tholen

**Affiliations:** Department of Structural Biology, Genentech Inc., South San Francisco, California 94080, USA

**Keywords:** RNA, snRNP, splice site, spliceosome, splicing

## Abstract

The spliceosome is a eukaryotic multimegadalton RNA–protein complex that removes introns from transcripts. The spliceosome ensures the selection of each exon-intron boundary through multiple recognition events. Initially, the 5′ splice site (5′ SS) and branch site (BS) are bound by the U1 small nuclear ribonucleoprotein (snRNP) and the U2 snRNP, respectively, while the 3′ SS is mostly determined by proximity to the branch site. A large number of splicing factors recognize the splice sites and recruit the snRNPs before the stable binding of the snRNPs occurs by base-pairing the snRNA to the transcript. Fidelity of this process is crucial, as mutations in splicing factors and U2 snRNP components are associated with many diseases. In recent years, major advances have been made in understanding how splice sites are selected in *Saccharomyces cerevisiae* and humans. Here, I review and discuss the current understanding of the recognition of splice sites by the spliceosome with a focus on recognition and binding of the branch site by the U2 snRNP in humans.

## INTRODUCTION

The excision of introns from transcripts of RNA polymerase II (RNAPII), including almost all protein-coding pre-mRNAs, is catalyzed by an RNA–protein complex called the spliceosome, which is several megadaltons large and involves at least 170 different proteins (Wahl et al. 2009).

The spliceosome undergoes dramatic rearrangements before, during, and after catalysis of the splicing reaction. The molecular rearrangements that lead to pre-mRNA splicing can be grouped into four phases: Assembly of the spliceosome, activation, catalysis, and disassembly. The spliceosome is assembled on each substrate RNA from smaller subunits, called the small nuclear ribonucleoproteins (snRNPs), as well as additional protein factors. All four phases of splicing heavily depend on nucleoside triphosphate (NTP)-dependent helicases to drive progression and ensure fidelity of splice site selection (Bortoli et al. 2021). The spliceosome has to identify and bind three specific positions on the pre-mRNA: the 5′ SS with a GU dinucleotide, the branch point adenosine (BP-A), and the 3′ SS with a YAG motif. The BP-A, together with surrounding nucleotides that are involved in base-pairing interactions with the U2 snRNA, form the branch site (BS). These conserved positions of the intron form part of the active center of the spliceosome. During the branching reaction, the 5′ SS G(+1) binds the BP-A (Galej et al. 2016; Wan et al. 2016). During exon ligation the 5′ SS GU and BP-A recognize and position the 3′ SS AG in the active site of the spliceosome by non-Watson-Crick base-pairing (Liu et al. 2017; Wilkinson et al. 2017). In order to ensure the selection of the correct splice site, each specific position is recognized several times (Wahl et al. 2009). A large network of splicing factors that differ for different classes of splice sites is involved in recruiting the U1 snRNP and the U2 snRNP, which bind to the 5′ SS and BS, respectively. This has so far prevented the outright prediction of many splice sites (Smith and Kitzman 2023).

Spliceosome assembly starts with the association of the U1 snRNP to the 5′ SS and the U2 snRNP to the BS (Shcherbakova et al. 2013). While base-pairing of U1 snRNA to the 5′ SS is ATP-independent, stable binding of the U2 snRNP requires ATP. The U2 snRNP is recruited to the transcript by splicing factors such as SF1 and U2AF which bind the BS and 3′ SS, respectively, and these are replaced by the U2 snRNP by ATP-driven helicases. The resulting spliceosomal complex containing the transcript bound to U1 and U2 snRNP is called the A complex or prespliceosome. The A complex binds the U6/U4.U5-tri-snRNP to form the pre-B complex. Splice sites can still change by several nucleotides during following recognition steps. For example, the 5′ SS can change as the pre-mRNA is transferred from U1 snRNA to the U6 snRNA (Kandels-Lewis and Séraphin 1993; Lesser and Guthrie 1993) and the 3′ SS could change as the C* spliceosome scans for a suitable AG dinucleotide that can catalyze the step II reaction (Dybkov et al. 2023).

Conformational and compositional changes of the spliceosome form the active site during the activation phase. The spliceosome catalyzes a two-step transesterification reaction, which results in the ligated exons and an excised intron lariat. Once the splicing reaction completes, the spliceosome is disassembled, releasing the products and snRNPs. The spliceosome may have some activity for hydrolyzing the 2′-5′ BS linkage and ligating the 5′ and 3′ end of the intron to form circular introns (Ares et al. 2024). After release from the spliceosome, the snRNPs have to undergo a recycling process before they can be reused for the assembly of another spliceosome.

More than 92% of all transcripts are spliced, generating a median 3.6 distinct isoforms in human tissues by selecting different splice sites in a process called alternative splicing (Wang et al. 2008; Tung et al. 2020). This enables the regulated production of a greater diversity of RNAs and proteins from a limited number of genes (Marasco and Kornblihtt 2023; Sinitcyn et al. 2023). Alternative splicing can regulate cellular processes by producing protein isoforms with different functions or cause the inclusion of premature termination codons which cause nonsense-mediated mRNA decay (NMD) of the transcript (Lewis et al. 2003). Thus, a strictly regulated machinery for the identification and removal of introns is required.

Splicing has been studied for many decades, with many of the pioneering discoveries made in the budding yeast *Saccharomyces cerevisiae* (Plaschka et al. 2019). This review focuses on splicing in humans. Although the mammalian and *S. cerevisiae* spliceosomes are highly similar in their working principle and in the structure of their core machinery, the mammalian spliceosome contains many additional splicing factors (Kastner et al. 2019). Most eukaryotes maintain two spliceosomes, a major spliceosome, and a minor spliceosome, which removes only about ∼0.4% of all introns, so-called U12-type introns (Sheth et al. 2006). Splice site consensus sequences are more conserved, and fewer splicing factors are known to affect primarily U12-type splicing, indicating that U12-type splice site recognition relies more heavily on sequence complementarity (Norppa et al. 2024).

Despite the centrality of pre-mRNA splicing in many biological processes, we do not have a complete picture of how introns are recognized by the spliceosome. Sequencing methods reveal the isoforms resulting from splicing, but it is not completely known how the spliceosome selects spice sites. Mutations in the SF3B1 subunit of the U2 snRNP cause selection of alternative splice sites and are implicated in diseases such as myelodysplastic syndrome (MDS) (Zhang et al. 2019a; Alsafadi et al. 2020). Several splicing inhibitors targeting the branchpoint adenosine pocket have failed in clinical studies, but continue to be of great interest for the treatment of tumors (Murphy et al. 2022).

Over the last decade, electron cryo-microscopy (cryo-EM) allowed the visualization of many structural rearrangements within the spliceosome and revealed the molecular mechanisms that ensure faithful excision of introns. Recent reviews have focused on the structures of spliceosomes (Kastner et al. 2019; Fica 2020; Wan et al. 2020; Tholen and Galej 2022) and the implications in health (Love et al. 2023; Marasco and Kornblihtt 2023; Rogalska et al. 2023). In this review, I focus on the recent discoveries on how BSs are identified and bound by the U2 snRNP.

### Most introns are spliced during transcription by the RNAPII

Exons are consistently short in humans (120 nt on average for internal exons), and introns are much longer with variable size (Movassat et al. 2019). Together with the observation of exon skipping when splice sites are mutated, it is expected that spliceosomes are usually assembled over exons (Berget 1995). Splicing may occur in proximity to the transcribing RNAPII. Many splicing factors also have a role in transcription regulation, showing crosstalk between the machineries (Alexander and Beggs 2010; Reimer et al. 2021). Microscopy and sequencing studies agree that cotranscriptional splicing varies strongly between introns and that introns of one transcript are spliced in a predefined order (Drexler et al. 2020). However, these studies disagree on the prevalence of exon or intron definition or cotranscriptional assembly. Single-molecule RNA FISH combined with expansion microscopy showed that splicing occurs generally posttranscriptionally, in a slow-moving zone surrounding the site of transcription (Coté et al. 2024). Nanopore sequencing of nascent RNA showed that <20% of introns are spliced before RNAPII has transcribed a further 1 kb (Drexler et al. 2020). However, other studies, such as cotranscriptional lariat sequencing (CoLa-seq), report that more than 90% of all introns perform the branching reaction before the downstream exon is transcribed, showing that exon definition may not be the default mode of splicing (Reimer et al. 2021; Zeng et al. 2022). Spliceosome components may even be physically bound to the transcription machinery, as seen in the cryo-EM structure of the U1 snRNP bound to RNAPII (Zhang et al. 2021a). The authors propose that U1 and U2 snRNP could be accommodated in the activated RNAPII transcription elongation complex and scan the nascent RNA during transcription.

### Recognition of the 5′ splice site by the U1 snRNP

U1 snRNP binding to the 5′ SS largely determines the 5′ end of the intron (Zhuang and Weiner 1986). Binding to the 5′ SS relies only partially on Watson–Crick base-pairing and may offer some guidance on how the U2 snRNP binds the even less complementary BS (Roca et al. 2013). Besides the conserved GU dinucleotide, a wide range of sequences with the consensus 5′-GUAUGU can bind U1 snRNA and form an 11 nt duplex (Sheth et al. 2006). Protein components of the U1 snRNP stabilize the formed helix and allow some mismatches (White et al. 2024). 5′ SS selection may be influenced by several variants of U1 snRNA that are expressed in human tissues and modifications like pseudouridylation (O'Reilly et al. 2013). It is possible that the initial 5′ SS recognition by the U1 snRNP may not be essential for some sequences, because under certain conditions splicing of some introns could proceed in nuclear extracts depleted of U1 snRNP (Crispino et al. 1996).

### Alternative splicing factors influence splice site selection

**FIGURE 1.**
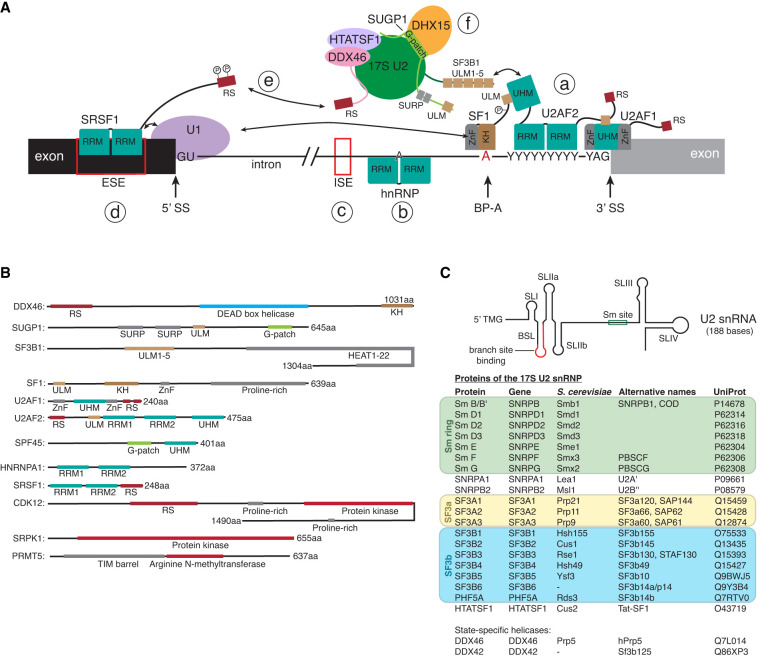
Components of the U2 snRNP and splicing factors involved in BS recognition. (*A*) Example components of the complex network of splicing factors involved in recognizing splice sites. (a) On most 3′ SS, the U2AF heterodimer and SF1 are the pioneering splicing factors (Ruskin et al. 1988; Zamore and Green 1989). These identify 3′ SS, BS, and the PPT, and recruit the 17S U2 snRNP, at least in part through the interaction of its UHM domains with the SF3B1 ULM motifs (Galardi et al. 2022). UHM-ULM interactions bring components of the splicing machinery together and are regulated by phosphorylation (Wang et al. 2013). (b) hnRNPs bind pre-mRNA and regulate splicing by blocking possible splice sites, recruiting splicing factors, or changing the structure of the pre-mRNA (Jones et al. 2022). (c) Intronic splicing enhancers (ISE) or (d) exonic splicing enhancers (ESE) can recruit proteins that enhance splicing. For example, the SRSF1 RRM domains bind ESE sequences while binding the U1 snRNP component SNRNP70 (U1-70K), recruiting it to 5′ SS on the pre-mRNA (Cho et al. 2011). The 5′ SS allows mismatches when base-pairing to U1 snRNP in every position, except the GU dinucleotide (Roca et al. 2013). (e) RS domain proteins (including SR proteins) form a network of protein–protein interactions that is regulated by phosphorylation. For example, the N-terminal RS domain of DDX46 is implicated in bridging U1 and U2 snRNP (Xu et al. 2004). (f) SUGP1 recruits DHX15 to the U2 snRNP and activates it through its G-patch domain (Zhang et al. 2023). SUGP1 also contains ULM and SURP domains, which are protein–protein interaction domains found only in splicing factors (Nameki et al. 2022). (*B*) Domain architecture of selected splicing factors that regulate BS selection. (*C*) The secondary structure and protein components of the 17S U2 snRNP. Depicted here are the components of the 17S U2 snRNP before they are bound to pre-mRNA. Not listed is Sm N, a substoichiometric component of the U2 snRNP that may replace Sm B/B’ in the U2 snRNP in certain tissues causing alternative splicing (Lee et al. 2014).

In mammals, the U1 snRNP and U2 snRNP rely on many factors in addition to the splice site sequence to find the correct splice sites (for a review with a list of known splicing factors, see Rogalska et al. [2023]). Many factors are not required for all introns and regulate the alternative splicing of transcripts. These alternative splicing factors rely on a variety of different mechanisms to recognize splice sites and recruit the U1 and U2 snRNP (Fig. 1A). Some splicing factors also regulate splice site selection after binding of the snRNP to the transcript (Damianov et al. 2024). The splicing factors are functionally grouped by the domains they contain, such as RS, helicase, SURP, KH, RNA recognition motif (RRM), U2AF homology domain (UHM), U2AF ligand motif (ULM), kinase, phosphatase, protein methyltransferase, and G-patch domains (Fig. 1B). For example, SR proteins are splicing factors that contain unstructured, arginine, and serine-rich RS domains that mediate interactions with proteins, mostly other RS domains (Wu and Maniatis 1993). Interactions between RS domains also change depending on the phosphorylation of the serine residues, creating a network of RS domain-containing proteins that regulate splicing (Stamm 2008). Reversible phosphorylation is required for splicing and may be used to keep track of the sequence of events (Mermoud et al. 1992). Inhibition of phosphatases in nuclear extract inhibits splicing at the pre-B/B stage (Zhan et al. 2018). UHM and ULM domains are part of many splicing factors and form another network of splicing factors. UHM domains have evolved from RRM domains to have reduced affinity to RNA and instead bind a ULM peptide consensus sequence (Loerch et al. 2014). Importantly, the U2 snRNP component SF3B1 has five ULMs with different affinities to different UHM domains that are likely involved in recruiting the U2 snRNP to BSs (Galardi et al. 2022). Another group of splicing factors is the highly abundant heterogeneous nuclear ribonucleoproteins (hnRNPs), which regulate splicing by recruiting splicing factors or by blocking splicing factors from binding sites on the transcript RNA (Huelga et al. 2012).

### Recognition of the BS and 3′ splice site by SF1 and U2AF

Most human BSs follow the consensus sequence YUNAY followed by the polypyrimidine tract (PPT), but these sequences are highly degenerate (Taggart et al. 2017). Selection of BS and 3′ SS are heavily intertwined, with the first AG 3′ after the BS usually selected as 3′ SS, even when another AG has promoted splicing (Zhuang and Weiner 1990). This indicates that the 3′ SS choice during the step II reaction is independent of initial recognition by splicing factors. In almost all introns, including the model substrates used for in vitro splicing assays, SF1 (mBBP) binds the BS, and U2AF binds SF1, the PPT, and 3′ SS of the intron (Zamore and Green 1989; Arning et al. 1996). U2AF is a heterodimer of U2AF2 (U2AF65), which binds the PPT, and U2AF1 (U2AF35), which binds the 3′ SS AG dinucleotide (Ruskin et al. 1988; Merendino et al. 1999). U2AF may not be necessary for some BSs (Kent et al. 2005), and depletion of SF1 in human cell lines does not affect splicing of all genes (Tanackovic and Krämer 2005). SPF45, which also binds SF3B1^ULM^ via its UHM domain, is essential for the U2AF-independent splicing of short introns (Fukumura et al. 2021). Some proteins, like PUF60 and RBM39, have UHM domains similar to U2AF2, which bind the U2AF1, SF1, and SF3B1 ULMs (Corsini et al. 2009). However, PUF60 and RBM39 act in a regulatory manner and do not replace U2AF2 (Hastings et al. 2007). Mutations of U2AF1, U2AF2, and RBM39 are associated with cancers and developmental effects, showing that alternative splicing factors are important regulators of development and cell homeostasis (Rogalska et al. 2023).

### The U2 snRNP binds the branch site

The U2 snRNP is a core component of the spliceosome important for BS recognition and whose subcomponents are involved in forming the active site. Before the U2 snRNP joins the spliceosome, it undergoes a long biogenesis process that starts with the transcription of the U2 snRNA by RNAPII, followed by a number of posttranscriptional modifications and association of proteins in both the nucleus and cytoplasm (van der Feltz and Hoskins 2019). The resulting 17S U2 snRNP contains the U2 snRNA bound to the heptameric Sm ring, SNRPA1 (Lea1/U2A′), SNRPB2 (Msl1/U2B″), as well as the subcomplexes SF3b and SF3a, and HTATSF1 (Fig. 1C). Additionally, the DEAD-box helicases DDX42 and DDX46 associate with 17S U2 snRNPs, but are mutually exclusive (Yang et al. 2023). The Sm ring, SNRPA1, and SNRPB2 form the stable core that remains assembled throughout the splicing cycle and is called the 12S U2 snRNP when dissociated from the spliceosome (Brosi et al. 1993; Lardelli et al. 2010). The U2 snRNP is released from the intron lariat spliceosome (ILS) without the SF3b and SF3a subcomplexes (Zhang et al. 2019b). The released U2 snRNP may be similar to the 12S U2 snRNP and has to undergo assembly of the 17S U2 snRNP again.

### Assembly of the 17S U2 snRNP

**FIGURE 2.**
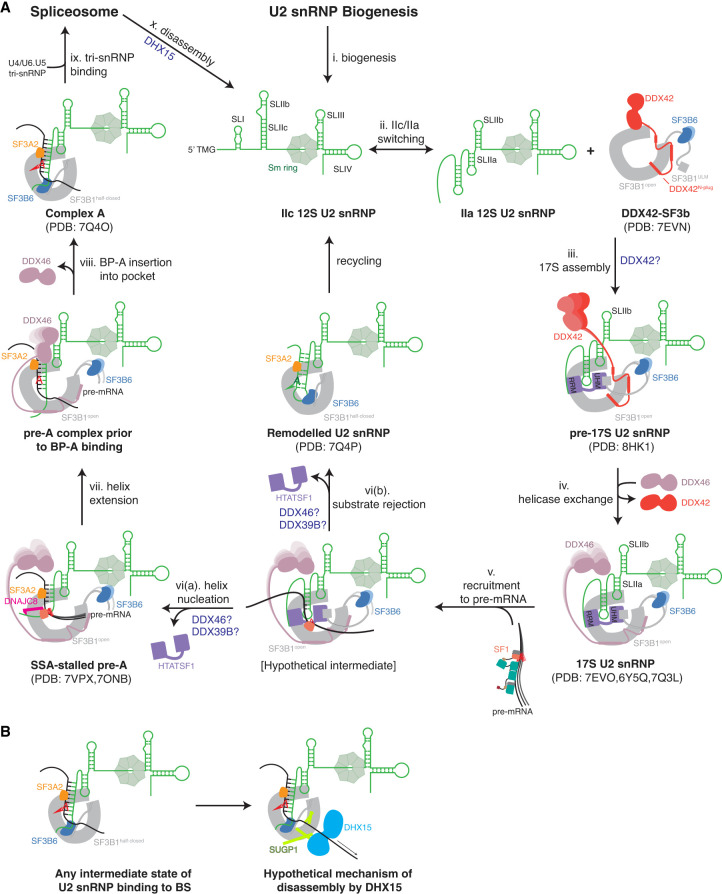
Schematic of BS binding by the U2 snRNP. (*A*) SF3a, SNRPA1, and SNRPB2 are omitted in this schematic. No structure of the U2 snRNP has so far shown stem–loop I, and therefore it is omitted as well. (i) The 12S U2 snRNP is created in a lengthy biogenesis process. (ii) The U2 snRNA can toggle between the IIc and IIa conformations (Rodgers et al. 2016). (iii) Association of the 12S U2 snRNP with the DDX42-bound SF3b complex and SF3a (Yang et al. 2023), possibly mediated by DDX42, forms the pre-17S U2 snRNP and stabilizes the IIa conformation (Yang et al. 2023). Through SF3B1^ULM1-5^, the free SF3b may already be bound to proteins like HTATSF1, which bind U2 snRNA (Tholen et al. 2022). (iv) DDX42 is displaced by DDX46 to form the DDX46-associated 17S U2 snRNP (Zhang et al. 2020, 2024; Tholen et al. 2022). (v) When the U2 snRNP is recruited to the transcript, the BSL may probe pre-mRNA for complementary sequences. [vi(a)] If complementary sequences are present, ATP hydrolysis by DDX46 removes HTATSF1, allowing the branch helix to extend, and the SF3A2 ZnF domain stabilizes it in a stalled pre-A complex (Cretu et al. 2021). [vi(b)] If DDX46 ATP hydrolysis and HTATSF1 removal occurs before formation of a toehold between BS and U2 snRNA, branch helix formation could compete with self-annealing of the U2 snRNA into a BMSL structure, which may be recycled into the 12S U2 snRNP (Tholen et al. 2022). (vii) The branch helix is fully formed in the pre-A complex, but the BP-A is not bound in its pocket. (viii) Once the bulged-out BP-A binds the pocket, SF3B1 transitions into a half-closed state, and DDX46 dissociates. SF3B6/p14 binds the U2 snRNA to SF3B1, stabilizing the branch helix. (ix) Complex A binds the U4/U6.U5 tri-snRNP to complete the assembly stage of the spliceosome. (x) Following catalysis of the splicing reaction, 12S U2 snRNP is released, allowing recycling of the U2 snRNP. (*B*) Possible mechanism of DHX15-mediated disassembly of A complexes or nonproductive intermediate states of BS binding (Zhang et al. 2023).

The U2 snRNA stem–loop II can exist in the IIa or IIc conformation (Fig. 2A, ii.; Hilliker et al. 2007). In yeast, the 17S-associated Cus2/HTATSF1 promotes interconversion between IIc and IIa (Rodgers et al. 2016), and mutations destabilizing IIa are more lethal when Cus2/HTATSF1 is mutated (Yan et al. 1998). The HTATSF1 U2AF homology motif (UHM) domain binds the N-terminal U2AF ligand motifs (ULMs) of SF3B1 (Loerch et al. 2019). This provides an essential binding interface between HTATSF1 and SF3B1 that may be established before 17S assembly. The highly conserved yeast homolog of HTATSF1, Cus2, binds U2 snRNA with a canonical RRM RNA-binding motif, while in humans, the other side of the RRM domain binds the SF3B1 HEAT repeats (Yan et al. 1998; Tholen et al. 2022). HTATSF1 thus forms a bridge between SF3B1 and U2 snRNA. In the 17S U2 snRNP, after integration of SF3b, SF3a, and HTATSF1, SF3b is bound tightly to the stem–loop IIa, indicating that SF3b stabilizes the IIa conformation (Zhang et al. 2020).

Once SF3b is bound, forming a 15S intermediate, SF3a can bind (Brosi et al. 1993). The SF3a complex bridges SF3b with the U2 snRNP core containing the Sm ring, SNRPA1 and SNRPB2 (Zhang et al. 2018). SF3A3 interacts with a stem–loop formed by the U2 snRNA in its BS binding region called the branchpoint-interacting stem–loop (BSL) (Zhang et al. 2020). HTATSF1 binds the BSL and stabilizes it both directly through interactions of the BSL with the linker and UHM domains, and indirectly by blocking the branch helix RNA binding site on the SF3B1 HEAT repeats (Tholen et al. 2022). The DEAD-box helicase DDX42 has been implicated in integrating SF3b into the U2 snRNP, because it is associated with both a free SF3b complex and a DDX42-bound 17S U2 snRNP (Fig. 2A, iii.; Will et al. 2002). DDX42 and DDX46 are mutually exclusive, so the DDX42-bound 17S U2 snRNP may be a precursor of the 17S U2 snRNP containing DDX46 (Fig. 2A, iv.; Yang et al. 2023). In the free SF3b complex, DDX42 is anchored to the SF3B1 HEAT repeats via its N-terminal domains (Yang et al. 2023). One of these domains is the N-plug, which occupies the same interface on the HEAT repeats as the downstream pre-mRNA following BS binding, SF3B1^630–828^. The DDX42 helicase domain may be loosely attached to the C-terminal HEAT repeats of SF3B1. The N-terminal acidic loop of DDX46 occupies an overlapping binding site to the DDX42 N-plug. Possibly, the N-plug of DDX42 prevents early or nonspecific binding of RNA on the N-terminal HEAT repeats of SF3B1. However, it has not been conclusively shown that DDX42 and DDX46 associate with the U2 snRNP sequentially, they could also work on different introns. Notably, no *S. cerevisiae* homolog of DDX42 is known.

### Stable binding of the U2 snRNP to the branch site requires ATP

Once the 17S U2 snRNP has been recruited to a pre-mRNA (Fig. 2A, v.), the U2 snRNP stably binds the pre-mRNA in an ATP-dependent reaction, and the U2 snRNA forms the branch helix with the substrate RNA (Wu and Manley 1989). Based on the functions of its *S. cerevisiae* homolog, Prp5, the ATPase activity of DDX46 likely has to remove HTATSF1 from SF3B1. In *S. cerevisiae*, the ATPase activity of Prp5/DDX46 is no longer essential in yeast if the interface of Cus2/HTATSF1 and Hsh155/SF3B1 is disrupted (Perriman and Ares 2000; Talkish et al. 2019). In this case, A complex formation is ATP-independent. This is the first of two major functions of DDX46 and the first ATP-dependent step of spliceosome assembly. Destabilizing mutations in the BSL likewise abrogate the need for Prp5/DDX46 ATPase activity (Perriman and Ares 2010). This can be explained by the function of HTATSF1 to stabilize the BSL before BS binding. Once the BSL is liberated from HTATSF1, U2 snRNA and pre-mRNA can nucleate a branch helix. Structures of spliceostatin A (SSA)-inhibited pre-A complexes show a partially formed branch helix stabilized by the SF3A2 ZnF domain through interactions with the pre-mRNA backbone [Fig. 2A, vi(a); Cretu et al. 2021; Zhang et al. 2024]. In these structures, SSA has covalently bound a cysteine in the BP-A pocket, blocking access for the pre-mRNA. Cretu et al. argued that the partially formed branch helix supports a mechanism whereby a toehold 5′ of the BP-A is initially formed and then extended in the 5′ direction. Zhang et al. (2024) discovered in their structure that the A complex-specific factor DNAJC8 is bound to SF3B1 HEAT repeats and SF1. SF1, likely bound to the BS, appears waiting to release the BS as the branch helix gets extended in the 3′ direction.

The formation of complex A is known to require the DECD box helicase DDX39B (UAP56, yeast Sub2) to remove SF1 and U2AF from the BS (Kistler and Guthrie 2001). It is possible that SSA has stalled branch helix formation right before DDX39B action. Unfortunately, the resolution in this part of the two cryo-EM maps of this state is low, preventing unambiguous interpretation (Cretu et al. 2021; Zhang et al. 2024). Interestingly, a short RNA oligo consisting of only the BS and a PPT can bind the 17S U2 snRNP in vitro to form a minimal A complex (Amin) independently of ATP (Query et al. 1997). An extension of the oligo in the 5′ direction by 18 nt causes ATP-dependence of its binding to the U2 snRNP. It is not known how a short RNA circumvents the ATP requirement.

### DDX46 may select BS sequences using a kinetic proofreading mechanism

Mutants of the *S. cerevisiae* DDX46 homolog Prp5 with lower ATPase activity permit suboptimal BS sequences (Xu and Query 2007). This may be explained by a kinetic proofreading mechanism, wherein a splicing-competent complex is only formed if the ATP hydrolysis occurs after the initial binding of the BS. If ATP hydrolysis and helicase activity occur before binding, the substrate is rejected. ATPase mutants with reduced activity allow substrates more time to bind and thus allow suboptimal substrates to bind. What happens if the pre-mRNA cannot form a branch helix is hinted at by the structure of the U2 snRNP after remodeling in the presence of ATP and absence of pre-mRNA (Tholen et al. 2022). In this structure, the U2 snRNA has self-annealed to form a branch helix-mimicking stem–loop [BMSL; Fig. 2A, vi(b)]. Like the branch helix, the BMSL bulges out an adenosine that enters the BP-A pocket, and the SF3B1 HEAT repeats are in a closed conformation when bound to the BMSL. Formation of the branch helix and BMSL may compete, and this would set a lower bound for the stability of the branch helix. The BMSL forms two patches of four and three base pairs, separated by looped-out RNA. While A complex-like structures have been produced using BS sequences with high complementarity (Tholen et al. 2022; Zhang et al. 2024), the BMSL shows how low-complementarity RNAs could still form a branch helix. It is possible that the BMSL is the result of the activity of DDX46 which destabilizes the BSL, which can then form the BMSL. I speculate that in cells, helicase activity is inhibited until an appropriate BS is presented to U2 snRNP, meaning the BMSL structure would rarely occur and would likely be recycled into the 12S U2 snRNP by DHX15.

### DDX46 also checks for proper formation of the branch helix

Appropriate substrates extend the branch helix until it is 15 nt long (Fig. 2A, vii). An intermediate state in which the bulged-out BP-A is not bound in its pocket may exist (Zhang et al. 2021b). It has been postulated that a second, ATP-independent function of DDX46 lies in the N-terminal half of Prp5/DDX46, which blocks splicing if the BP-A is not bulged out (Liang and Cheng 2015). This was confirmed by the structure of a yeast pre-A complex without a BP-A (Zhang et al. 2021b). The stalled structure was likely enabled by the much more stringent requirement for conserved BS sequences in *S. cerevisiae*. In this complex, the RecA1 helicase domain of Prp5/DDX46 is placed between BSL and SLIIb. Likely, the following structural rearrangements in SF3B1 caused by binding of BP-A into the pocket would release the N-terminal domains of DDX46 and allow the U4/U6.U5 tri-snRNP to bind the A complex.

A similar approach of mutating the BP-2 position to stall the A complex was used to obtain a low-resolution model of the yeast A complex (Plaschka et al. 2018). In this sample, Prp5/DDX46 was present in very low amounts, so it was likely dissociated, but low resolution (>10 Å) prevents interpretation of this part of the cryo-EM map, and it remains unclear how this complex is stalled.

Once the BP-A binds the pocket formed by SF3B1 and PHF5A, the SF3B1 HEAT repeats close around the pocket as a hinge into a half-closed state, and DDX46 dissociates (Fig. 2A, viii; Tholen et al. 2022). The structure of this A complex-like state was obtained by incubating purified 17S U2 snRNP in vitro with a short RNA oligo with a BS, similar to the Amin complex (Query et al. 1997). The U2 snRNA 5′ end emerging from the branch helix is bound between SF3B1 and SF3B6^RRM^ (Tholen et al. 2022). This stabilization of the branch helix may be necessary to allow the formation of a branch helix for sequences without complementarity and explains why there is no SF3B6 homolog in *S. cerevisiae*.

### The DEAH box helicase DHX15 disassembles aberrant spliceosomes

The helicase DHX15 is known to be involved in the disassembly of the ILS spliceosome after release of the mRNA product (Arenas and Abelson 1997). Now, data is emerging that DHX15 is also a guardian of proper BS selection. The Amin complex formed by the 17S U2 snRNP with a short BS oligo without ATP is disassembled in nuclear extract in the presence of ATP or GTP (Newnham and Query 2001; Maul-Newby et al. 2022). DEAH box helicases can use GTP, unlike DEAD box helicases (Bortoli et al. 2021). Proteomic studies show that the U2 snRNP contains the DEAH box helicase DHX15 (Prp43) and DHX15 has a known function in the disassembly of aberrant spliceosomes, therefore DHX15 likely catalyzes the disassembly of the nonproductive Amin complex (Fourmann et al. 2013; Maul-Newby et al. 2022). Another hint at this function of DHX15 has emerged from the investigation of cancer-associated mutations in the SF3B1 HEAT repeats (Zhang et al. 2019a). These were found to disrupt the interaction with SUGP1, which contains a G-patch domain, an activator of DEAH box helicases. Mutations in SUGP1 or DHX15 recapitulate the aberrant use of upstream BS and 3′ SS also caused by SF3B1 mutations (Zhang et al. 2022). SUGP1 and DHX15 were also found to work together to enhance splicing fidelity, promote strong BS, and cause hypersensitivity to the splicing inhibitor pladienolide B (Alsafadi et al. 2020; Zhang et al. 2022; Beusch et al. 2023). Meanwhile, GPATCH8 deletion reverses some of the SF3B1 mutant splicing defects and also binds DHX15 with its G patch domain, indicating that SUGP1 and GPATCH8 have opposing functions (Benbarche et al. 2024).

SUGP1 binding to DHX15 requires its ULM domain, and, surprisingly, deleting the G-patch enhances the coprecipitation of SUGP1 with DHX15 (Feng et al. 2023). This implies that SUGP1 must be recruited to DHX15 through another protein which contains a UHM domain, and then the G-patch domain activates helicase activity, which dissociates the complex. In the AlphaFold2-predicted model of SF3B1:SUGP1:DHX15, SUGP1 is within the range of SF3B1^ULM1-5^ (Zhang et al. 2023). Considering the low specificity of UHM-ULM interactions, it can be speculated that SF3B1^ULM^ may antagonize DHX15 recruitment.

DHX15 disassembles the ILS spliceosome by translocating along the U6 snRNA in the 3′–5′ direction (Toroney et al. 2019). It is not clear whether DHX15 pulls on pre-mRNA or U2 snRNA in early splicing complexes before association of the U6 snRNA. DHX15 could move the pre-mRNA while it is bound to the U2 snRNP, thereby scanning the pre-mRNA for suitable BS, or it could pull on either to dissociate the RNAs. In the AlphaFold2-predicted model, DHX15 was proposed to be located close to the downstream pre-mRNA binding site on SF3B1^HEAT^ (Fig. 2B). This would suggest pre-mRNA as the DHX15 substrate as U2 snRNA would have to be fully extended from SLIIa for the 5′ end to reach this far. This may explain the upstream shift of BS with SF3B1 mutants: DHX15 is not recruited, meaning it does not pull in the 3′ direction, causing the branch helix to stay upstream. In healthy cells, DHX15 would pull on the pre-mRNA until a more stable BS is found.

Several mechanisms have been proposed for DHX15-mediated quality control (Feng et al. 2023). One mechanism is kinetic proofreading (Hopfield 1974), where activation of DHX15 disassembles the spliceosome after a certain amount of time (Feng et al. 2023). Kinetic proofreading by DEAH helicases to supervise the splicing process may be a common mechanism (Semlow and Staley 2012). During formation of the branch helix, binding sites for G-patch proteins like SUGP1 could be available, and these recruit and activate DHX15. BS that take longer to form a branch helix would have a higher probability of disassembly by DHX15, meaning slower binding BS are less likely to form spliceosomes.

### Open questions

In the last few years, a wave of cryo-EM structures has propelled the field forward. However, the recent cryo-EM structures have often been obtained using assembly and purification protocols that were already established over the last decades and easily adapted for cryo-EM. The transitions between these biochemically defined splicing complexes likely proceed via multiple intermediate states, but these have never been captured. Now, new approaches have to be developed to stall these intermediate states of BS binding. Novel purification methods, such as that described by Damianov et al. (2024), could give insight into intermediate stages of BS binding. They isolated parts of a chromatin-associated A complex containing RBM5 and RBM10, which may regulate selection of the bound BS and thereby exon inclusion.

Many questions remain about the function and interplay of the four U2-associated helicases DDX39B, DDX42, DDX46, and DHX15. Figuring out how these work mechanistically is a major goal of future investigation. Structures of DHX15 acting on the U2 snRNP are not available. The structures of 17S U2 snRNP and of A complexes do not visualize DDX46's proposed activity on the BSL. Obtaining an intermediate structure that reveals the order of substrate binding, DDX39B & DDX46 helicase activities, and HTATSF1 removal could explain currently contradictory biochemical data (Liang and Cheng 2015). Additionally, previous structures were determined with BS sequences that are highly complementary to the U2 snRNA. The structure of the BMSL in the ATP-remodeled U2 snRNP hints at how the U2 snRNP accommodates mismatches in the branch helix by looping out long stretches of RNA. Future structures could show if this is a general mechanism and how the branch helix may be stabilized.

The convoluted effects of many different alternative splicing factors influence the selection of each splice site. One exciting development for identifying subsets of introns that rely on certain splicing factors is the recent improvement in machine learning/AI algorithms in tandem with RNA sequencing methods that provide data for these algorithms (Smith and Kitzman 2023). These algorithms could allow the high-throughput classification and analysis of splice sites. This would not just improve the predictions of splice sites, but also the targeted biochemical investigation of unique splice sites and the roles of splicing factors.
